# Microbead‐based synthetic niches for in vitro expansion and differentiation of human naïve B‐cells

**DOI:** 10.1002/btm2.10751

**Published:** 2025-01-17

**Authors:** Pearlson Prashanth Austin Suthanthiraraj, Sydney Bone, Kyung‐Ho Roh

**Affiliations:** ^1^ Department of Chemical and Materials Engineering The University of Alabama in Huntsville Huntsville Alabama USA; ^2^ Biotechnology Science and Engineering Program The University of Alabama in Huntsville Huntsville Alabama USA; ^3^ Materials Science Program The University of Alabama in Huntsville Huntsville Alabama USA

**Keywords:** B‐cell, Adoptive cell therapy, CD40L, Artificial germinal center, Plasma cell, Microbeads

## Abstract

As the prospect of engineering primary B‐cells for cellular therapies in cancer, autoimmune diseases, and infectious diseases grows, there is an increasing demand for robust in vitro culture systems that effectively activate human B‐cells isolated from peripheral blood for consistent and efficient expansion and differentiation into various effector phenotypes. Feeder cell‐based systems have shown promise in providing long‐term signaling for expanding B‐cells in vitro. However, these co‐culture systems necessitate more rigorous downstream processing to prevent various feeder cell‐related contaminations in the final product, which limits their clinical potential. In this study, we introduce a microbead‐based CD40L‐presentation platform for stable and consistent activation of human naïve B‐cells. By employing a completely synthetic in vitro culture approach integrating B‐cell receptor, CD21 co‐receptor, toll‐like receptor (TLR‐9), and cytokine signals, we demonstrate that naïve B‐cells can differentiate into memory B‐cells (IgD‐CD38‐/lo + CD27+) and antibody‐secreting cells (IgD‐CD38++CD27+). During this process, B‐cells underwent up to a 50‐fold expansion, accompanied by isotype class switching and low levels of somatic hypermutation, mimicking physiological events within the germinal center. The reproducible generation of highly expanded and differentiated effector B‐cells from naïve B‐cells of multiple donors positions this feeder‐free in vitro synthetic niche as a promising platform for large‐scale production of effector B‐cell therapeutics.

**Translational Impact Statement**

Current in vitro B‐cell activation protocols using feeder cells and serum hinder clinical manufacturing. We present a microbead‐based, feeder‐free, and serum‐free system for converting naïve B‐cells into memory and antibody‐secreting cell phenotypes. This method uses finely tuned CD40L signals by microbeads and soluble factors for B‐cell receptor, CD21, TLR‐9, and cytokine signals, resulting in isotype switching and substantial cell expansion. This system's universal activation of naïve B‐cells from multiple donors shows potential for significantly advancing the clinical translation of B‐cell therapies.

## 1 INTRODUCTION

B‐cells become a critical arm of adaptive immunity, along with T‐cells. Mainly, B‐cells are responsible for humoral immunity by secreting antigen‐specific antibodies. Upon detection of the pathogen by the B‐cell receptor (BCR), the naïve B‐cells that circulate in the blood and lymph migrate into the secondary lymphoid organs, where they form specialized microenvironments called germinal centers (GCs).^1^, ^2^ Within GCs, the naïve B‐cells receive complex and dynamic signals mainly from follicular helper T‐cells and undergo rapid proliferation, class switch recombination (CSR), and affinity maturation via somatic hypermutation (SHM).^1^ These B‐cells also differentiate into the effector B‐cell phenotypes, specifically memory B‐cells and antibody‐secreting plasma cells. In addition to this T‐dependent mechanism, B‐cells can also be activated by T‐independent mechanisms, for example, by detecting pathogenic molecules such as the polysaccharides of bacterial cell walls via toll‐like receptors (TLRs).^3^, ^4^

With the tremendous clinical success of adoptive cell transfer therapies such as chimeric antigen receptor T‐cells in recent decades, engineering B‐cells has drawn significant interest to leverage the unique abilities of B‐cells as cellular therapeutics to treat infectious diseases, cancers,^5^, ^6^, ^7^ autoimmune diseases, and other protein deficiencies.^8^ For example, the immunoglobulin (Ig) genes of somatically mutated B‐cells were successfully edited to improve neutralizing specificity against human immunodeficiency virus (HIV), respiratory syncytial virus, influenza virus, and Epstein–Barr virus.^9^, ^10^, ^11^ Furthermore, gene editing has also shown promise in inducing human primary B‐cells to differentiate into plasma cells that secrete specific therapeutic proteins after homing to the bone marrow in humanized mice.^12^ Despite success in preliminary trials, the genetically engineered B‐cells have not yet been successfully translated to clinics. One of the primary roadblocks that hamper such translation is the lack of a universally applicable in vitro activation mechanism for B‐cells. For successful translation of engineered B‐cells into clinical applications, the primary B‐cells must be expanded ex vivo to sufficient numbers before or after genetic engineering while generating desired effector phenotypes.

In fact, many in vitro B‐cell activation approaches have been previously explored by employing both T‐dependent and T‐independent activation mechanisms. A majority of these protocols for T‐dependent mechanism have involved B‐cell activation using feeder cells transfected with the CD40 ligand, one or more of the interleukins IL‐2, IL‐4, IL‐10, IL‐21, and the B‐cell activation factor (BAFF).^13^, ^14^, ^15^, ^16^, ^17^ These feeder‐based activation systems have successfully expanded human B‐cells from healthy donors^17^ or from cancer patients, demonstrating their potential use for cancer immunotherapy^13^ as antigen presenting cells to activate autologous T‐cells in vitro.^16^, ^17^, ^18^ Some utilized autologous T‐cells to expand the B‐cells while avoiding the use of exogenous feeder cells.^19^, ^20^ In order to effectively induce T‐dependent activations without feeder or autologous cells, efforts have been driven toward engagement of the CD40 receptor using agonistic anti‐CD40 monoclonal antibodies^21^, ^22^, ^23^, ^24^ or recombinant CD40L.^25^ Recently, using tetrameric CD40L, human naïve B‐cells (CD19 + CD27‐IgG‐) were successfully activated to undergo CSR and differentiation into plasma cells.^26^ For murine splenic B‐cells, recombinant CD40L presented on the surface of the microbeads together with IL‐4 and BAFF efficiently induced GC‐like reactions.^27^ Meanwhile, B‐cell activation via T‐independent mechanisms, such as the activation of the BCR and TLRs, have also been explored with or without CD40L activation and the interleukins.^28^, ^29^, ^30^ In these studies, anti‐immunoglobulin antibodies targeting IgM or a mixture of anti‐IgM, IgG, and IgA antibodies were used to activate BCR, while CpG oligodenucleotide (ODN), a synthetic TLR‐9 agonist, was also employed. In general, synergy between multiple activation mechanisms results in better B‐cell differentiation outcomes when compared to standalone activation.^15^, ^27^, ^31^, ^32^, ^33^

Despite the numerous efforts, we still do not have a commonly accepted culture protocol for effective expansion and differentiation of human naïve B‐cells to yield effector B‐cell phenotypes in vitro for clinical translation into B‐cell therapy due to several complications. Both the feeder‐based and feeder‐free B‐cell cultures in the previous literature suffer from certain limitations for clinical applications. The feeder cells require frequent irradiation and/or chemical treatment to minimize feeder cell growth^34^ and intrinsically add significant burdens on the cell purification and separation steps in the manufacturing process of B‐cell therapeutics. It might also require the use of proteolytic and/or collagenolytic enzymes to detach primary B‐cells from feeder cells, which could degrade certain cell surface receptors and ligands, thus limiting their downstream applications.^35^ On the other hand, most feeder‐free cultures use serum, which carries the intrinsic risk of lot‐to‐lot variability, exogenous contamination, and infection. The next hurdle that prohibits the direct translation of some of the previous literature is related to the fact that the B‐cell culture outcomes vary greatly depending upon the purity of the starting cell populations. In many published culture conditions, CD19+ B‐cells were used on day‐0 as the starting B‐cells. But this population may consist of not only naïve B‐cells but also memory B‐cells and double negative (CD27‐IgD‐) memory B precursors. By virtue of a relatively lower signaling threshold, these pre‐activated and differentiated B‐cells can readily expand and further differentiate into plasma cells, thus creating a bias in the culture outcome.^36^, ^37^ Such heterogeneity in starting B‐cell populations from different donors contributes to wide variations in the overall culture outcomes.

So, it is imminently needed to develop cost‐effective and scalable in vitro B‐cell activation protocols that can be easily integrated into current cell manufacturing practices to support the high‐volume manufacturing of cell therapies against infectious diseases, cancers, and autoimmune diseases. In this paper, we used highly pure (>95% purity) human naïve B‐cells (IgD + CD27‐) isolated from peripheral blood mononuclear cells (PBMCs) as the most standardized starting B‐cell population from the most abundant clinically relevant source. We tested and employed the feeder‐free and serum‐free conditions, considering their best translational potential. Mimicking the membrane‐bound CD40L signaling presented by T‐cells within the physiological GCs, we presented CD40L on the surface of iron oxide microbeads while the BCR and TLR signals were added as soluble factors. We first characterized B‐cell activation via microbead CD40L together with BAFF and IL‐4 via imaging and compared the kinetics of the morphological changes in individual activated B‐cells and their clustering. Next, we studied the effect of the sequential addition of interleukins IL‐2, IL‐10, and IL‐21 in efficiently driving B‐cells towards the GC B‐cells and expanding and differentiating them into the effector phenotypes. For the final B‐cells harvested from selected culture conditions, we examined the section of antibodies via ELISA and further investigated the frequency of SHM and CSR via BCR sequencing. Our results indicate that the synthetic niche created by the microbead‐based CD40L presentation and the synergistic use of BCR, TLR‐9, and interleukin signals with temporal regulation in quantity and quality enables effective expansion and sequential differentiation of human naïve B‐cells into memory B‐cells and ASCs.

## 2 RESULTS

### 2.1 Feeder‐free CD40L presentation and early activation of B‐cells

**FIGURE 1:**
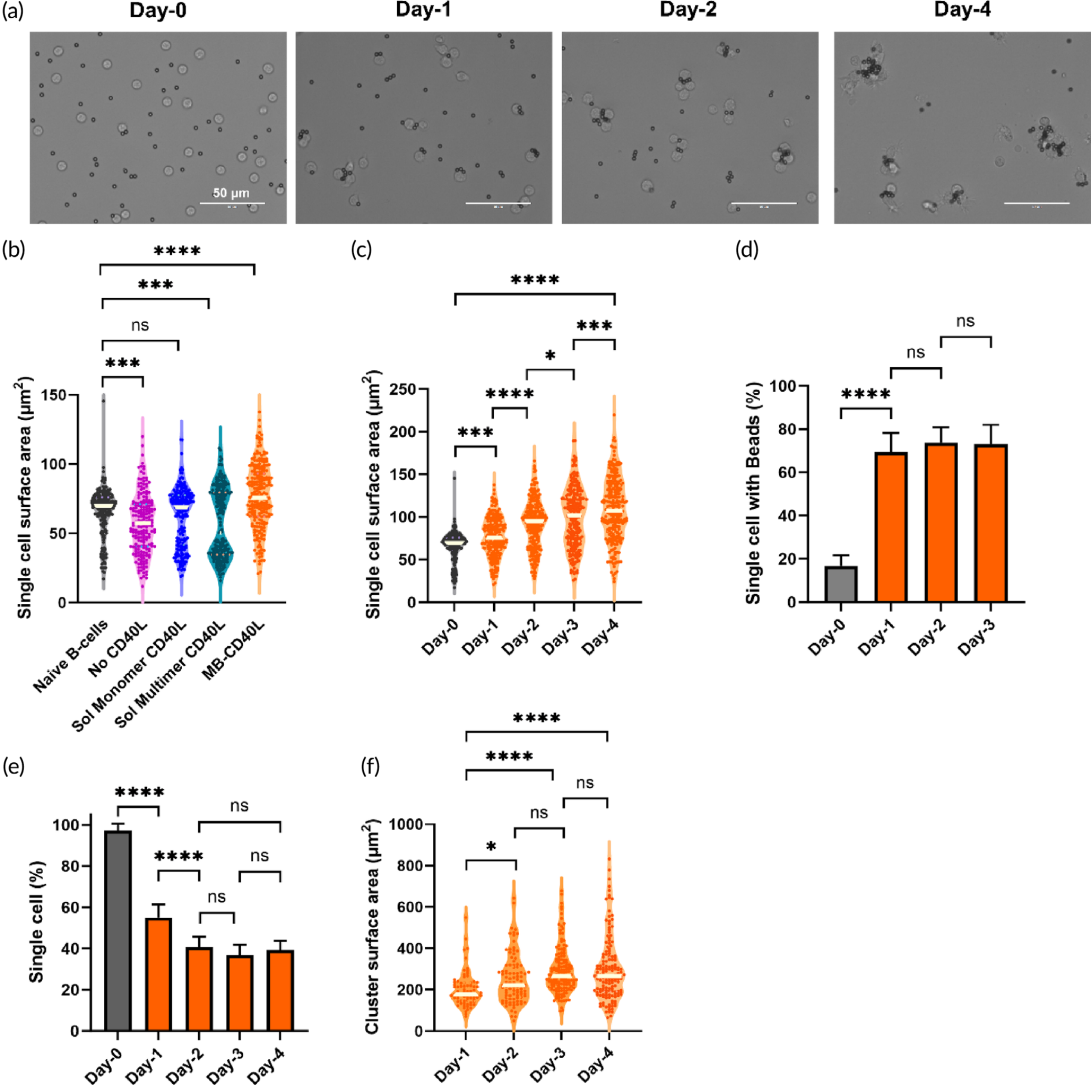
Characterization of early B‐cell activation using feeder‐free CD40L presentation. (a) Optical microscopy images (63×) of human naïve B‐cells on day‐0 and activated up to day‐4 in the presence of microbeads presenting CD40L (MB‐CD40L), IL‐4, and BAFF. (b) Plot comparing the single cell surface area of naïve B‐cells with the B‐cells after 18 h of activation via indicated methods of CD40L presentation. Each data point in the violin plot represents one single B‐cell. (c) Plot tracing the change in the single cell surface area of naïve B‐cells with that of the B‐cells activated by MB‐CD40L up to day‐4. (d) Plot comparing the percentage of single cells associated with MB‐CD40L on day‐0 (1 h) up to day‐3. (e) Plot comparing the percentage of single cells (with and without microbeads) on day‐0 with that after activation by MB‐CD40L up to day‐4. (f) Plot tracing the change in the surface area of B‐cell clusters formed by activating naïve B‐cells with MB‐CD40L from day‐1 up to day‐4. Statistical analysis was conducted by applying two‐way ANOVA with Tukey's multiple comparisons (***p ≤ 0.001; ****p ≤ 0.0001; ns *p* > 0.05).

Naïve B‐cells undergo changes in shape and dimensions upon activation. GC B‐cells are generally larger than the naïve B‐cells and exhibit highly polarized, probing morphology, having extended dendrites and uropod structures.^1^ So, we first compared the effectiveness of various feeder‐free methods of CD40L presentation in the activation of naïve B‐cells by monitoring the changes in the B‐cell shape and/or dimensions. We used two soluble CD40L groups, namely monomeric and multimeric forms, and CD40L presented on the surface of iron oxide microbeads (MB‐CD40L). In all groups, the CD40L concentration was maintained constant (100 ng/mL), and common factors of IL‐4 and BAFF were used. Based on measuring the surface area of at least 200 individual B‐cells, the naïve B‐cells isolated from PBMCs have a surface area of ~70 μm^2^ (Figure 1b). After 18 h of incubation, in the negative control group with IL‐4 and BAFF alone, that is, no CD40L activation, the average cell surface area decreased significantly, probably due to increased cell death. In both soluble CD40L conditions, two distinct lobes, indicative of two sub‐populations, were observed in the violin plot. It seems that soluble CD40L activation was ineffective, at least for an unknown subset population among naïve B‐cells (IgD + CD27‐). While any changes induced by the monomeric CD40L were not significantly different (*p* = 0.4818) from overall naïve B‐cells, the divergent change in the cell surface area was significantly different for the soluble multimeric CD40L (*p* = 0.0006). In contrast, MB‐CD40L resulted in a statistically significant increase in the overall cell surface area (*p* < 0.0001). As shown in Figure 1c, the B‐cell activation by MB‐CD40L continued to induce significant changes in the cell dimensions each day, resulting in a ~ 1.5× increase in the cell surface area by day‐4 relative to the naïve B‐cells. Furthermore, these activated B‐cells also displayed an elongated morphology, with dendrite‐like protrusions extending from the periphery of individual B‐cells.

As also observed in Figure 1a, this effective initial activation of naïve B‐cells is accompanied by rapid association of MB‐CD40L on individual B‐cells. Based on the analyses of at least 600 single cells, approximately 17% of single cells are associated with at least one MB‐CD40L during the initial 1 h of incubation, and that number significantly grew to 70% by day‐1 (Figure 1d). Each single cell was associated with up to 5 microbeads (Supplementary Figure 1). In addition, the three‐dimensional nature of CD40L presentation on the spherical surface of the microbeads enabled the activated B‐cells to grow into semi‐3D cellular clusters (Figure 1a). The number of cells in each cluster increased from just 2 cells on day‐1, to 3–4 cells per cluster on day‐2 and 6–8 cells by day‐4. Based on the analyses of at least 1500 cells, the percentage of cells that remain as a single cell (without being part of a cluster) was reduced to ~50% and ~ 40% by day‐1 and day‐2, respectively (Figure 1e).

By the combined effects of increased dimension of single cells and continued cell clustering, the surface area occupied by cell clusters measured by microscopy also increased (Figure 1f). Overall, these observations up to day‐4 confirm that the feeder‐free presentation of CD40L on the surface of microbeads (MB‐CD40L) is effective for early activation of B‐cells.

### 2.2 Effects of interleukins on expansion and differentiation of human naïve B‐cell into effector phenotypes

**FIGURE 2:**
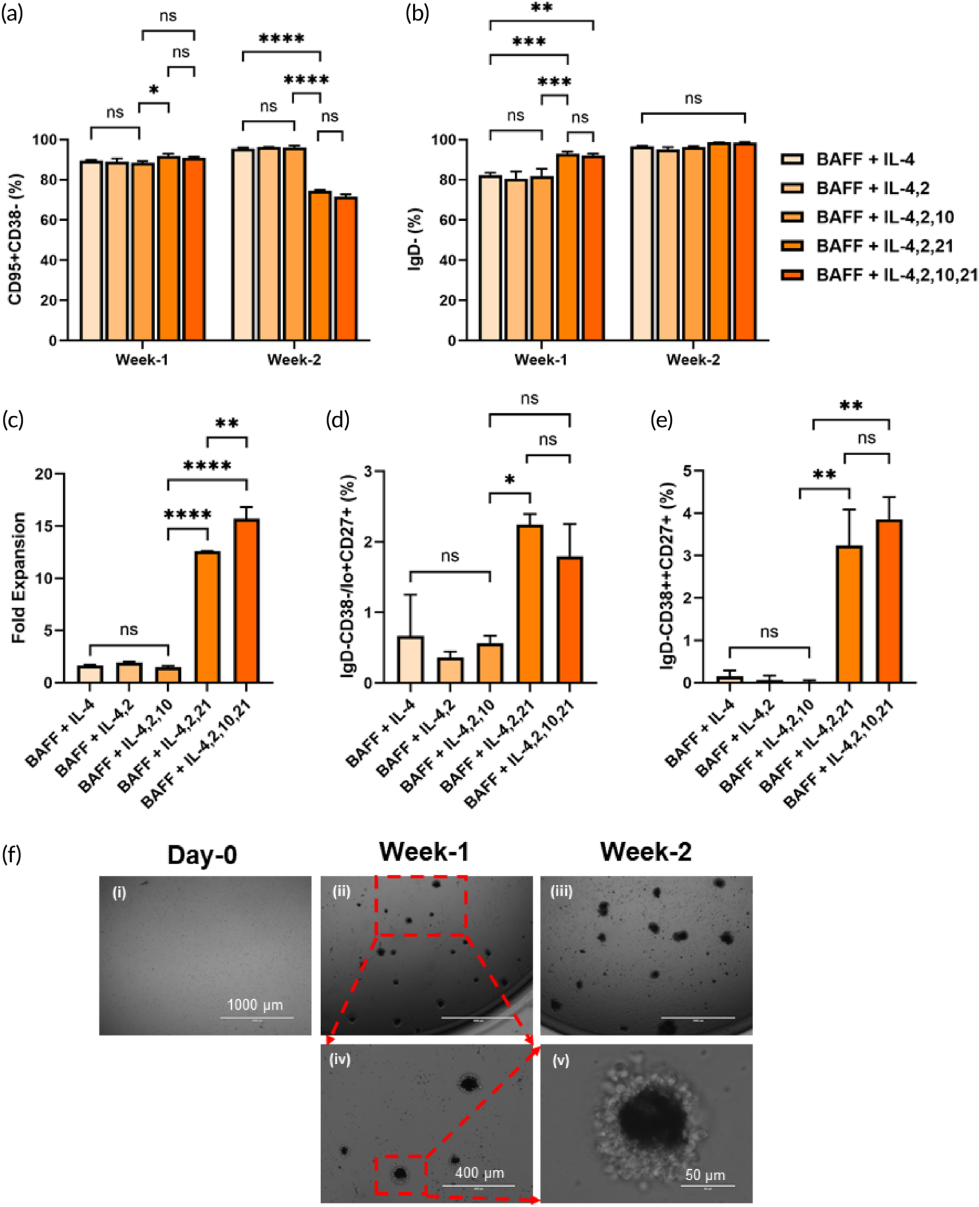
Effects of interleukins on activation of human naïve B‐cells using MB‐CD40L. Plot comparing the yield of indicated phenotypes (a, b, d, and e) and fold expansions at the end of week‐1 and week‐2 (a and b) or week‐2 (c)–(e). (a) GC B‐cells (CD95 + CD38‐); (b) IgD‐ B‐cells; (c) fold expansion of live activated B‐cells compared to the number of seeded cells on day‐0; (d) memory B‐cells (IgD‐CD38‐/lo + CD27+); and (e) antibody‐secreting cells (ASCs) (IgD‐CD38++CD27+). Data were acquired from at least duplicates for each group each day and presented as mean + standard deviation. Statistical analysis was conducted by applying two‐way ANOVA with Tukey's multiple comparisons for comparing week‐1 and week‐2 data and one‐way ANOVA with Tukey's multiple comparisons for comparing the end‐point results (*p ≤ 0.05; **p ≤ 0.01; ***p ≤ 0.001; ****p ≤ 0.0001; ns *p* > 0.05). (f) Microscopy images acquired at 4× magnification of (i) human naïve B‐cells seeded into a 96‐well plate on day‐0 and activated by MB‐CD40L up to (ii) week‐1 and (iii) week‐2 in the presence of IL‐4, BAFF, IL‐2, IL‐10, and IL‐21 showing the growth of B‐cells and their clusters. (iv) 10×and (v) 63× magnification images. Darker contrasts originate from the microbeads, and lighter contrasts originate from the B‐cells.

In addition to IL‐4 and BAFF, the interleukins IL‐2, IL‐10, and IL‐21 are also known to play key roles at various stages of B‐cell activation and differentiation.^15^, ^29^, ^38^ We studied the effects of these interleukins on the activation of naïve B‐cells using MB‐CD40L. While keeping IL‐4 and BAFF as the common factors, we sequentially added interleukins IL‐2, IL‐10, and IL‐21. As expected from the observations described in the previous section, the activation induced by MB‐CD40L with IL‐4 and BAFF was sufficient to differentiate the majority (>80%) of the naïve B‐cells (CD95‐CD38lo/+) to GC B‐cell phenotypes (CD95 + CD38‐) by day‐7 (Figure 2a). During the second week of culture, under conditions containing IL‐21 with or without IL‐10, a significant decrease in the yield of GC B‐cell phenotype was observed (*p* < 0.0001), which indicates that the addition of IL‐21 further differentiated the GC B‐cells into effector phenotypes by the second week of culture.

As another early activation marker, we measured the downregulation of IgD as an initial indication of CSR. Similar to differentiation into GC B‐cell phenotypes, MB‐CD40L with IL‐4 and BAFF with or without the addition of IL‐2 and/or IL‐10, IgD was downregulated in ~80% of the activated B‐cells at the end of week‐1, and the further addition of IL‐21 improved this yield to ~95% (Figure 2b). With continued activation by MB‐CD40L, the overall yield of IgD‐ B‐cells increased to >95% by week‐2 for all groups.

Although MB‐CD40L with IL‐4 and BAFF successfully induced differentiation of naïve B‐cells into GC B‐cell phenotypes and downregulated IgD, it was insufficient to expand B‐cells effectively (Figure 2c). The overall fold expansion was limited to less than 2‐fold even after 2 weeks of culture, and further addition of IL‐2 and/or IL‐10 did not make a significant change either (Figure 2c). The addition of IL‐21 drastically improved B‐cell expansion (*p* < 0.0001) to at least 10‐fold, which further increased to 15‐fold with the addition of IL‐10 (*p* < 0.001) (Figure 2c). Figure 2f shows the microscopy images of B‐cells activated via MB‐CD40L in the presence of IL‐4, BAFF, IL‐2, IL‐10, and IL‐21, visibly showing the robust expansion of B‐cells. As they grew in number, the activated B‐cells and MB‐CD40L formed clusters of approximately 100 μm in diameter by the end of week‐1, and the size increased to approximately 300 μm in diameter by the end of week‐2.

As the canonical outcomes of the physiological GC reaction, the activated B‐cells would differentiate into memory B‐cells (IgD‐CD38‐/lo + CD27+) and antibody‐secreting cells (ASCs) (IgD‐CD38++CD27+). Similar to B‐cell expansion, IL‐4, BAFF, and further addition of IL‐2 and/or IL‐10 were insufficient to differentiate B‐cells toward these effector phenotypes by week‐2. However, the addition of IL‐21 significantly improved (*p* < 0.001) the yield of both memory B‐cells (Figure 2d) and ASCs (Figure 2e). The contributions from IL‐2 or IL‐10 seemed nominal (*p* > 0.05).

Indeed, it was previously shown that CD40L activation in the presence of either IL‐4 or IL‐21 alone was sufficient to differentiate CD19+ B‐cells to memory B‐cells and plasma cells.^38^ However, the CD19+ B‐cells in the blood consist of not only naïve B‐cells but also many other subsets, including transitional B‐cells, memory B‐cells, double negative B‐cells, and regulatory B‐cells, thus may not be ideal as a starting population to study the activation of naïve B‐cells. Here, we tested whether MB‐CD40L with IL‐4 or IL‐21 alone can induce differentiation of naïve B‐cells into effector B‐cell phenotypes by limiting the starting B‐cell population more strictly to naïve B‐cells (>95% CD19 + IgD + CD27‐). For comparison, we also included a condition with all interleukins tested above, namely IL‐4, IL‐2, IL‐10, IL‐21, and BAFF.

**FIGURE 3:**
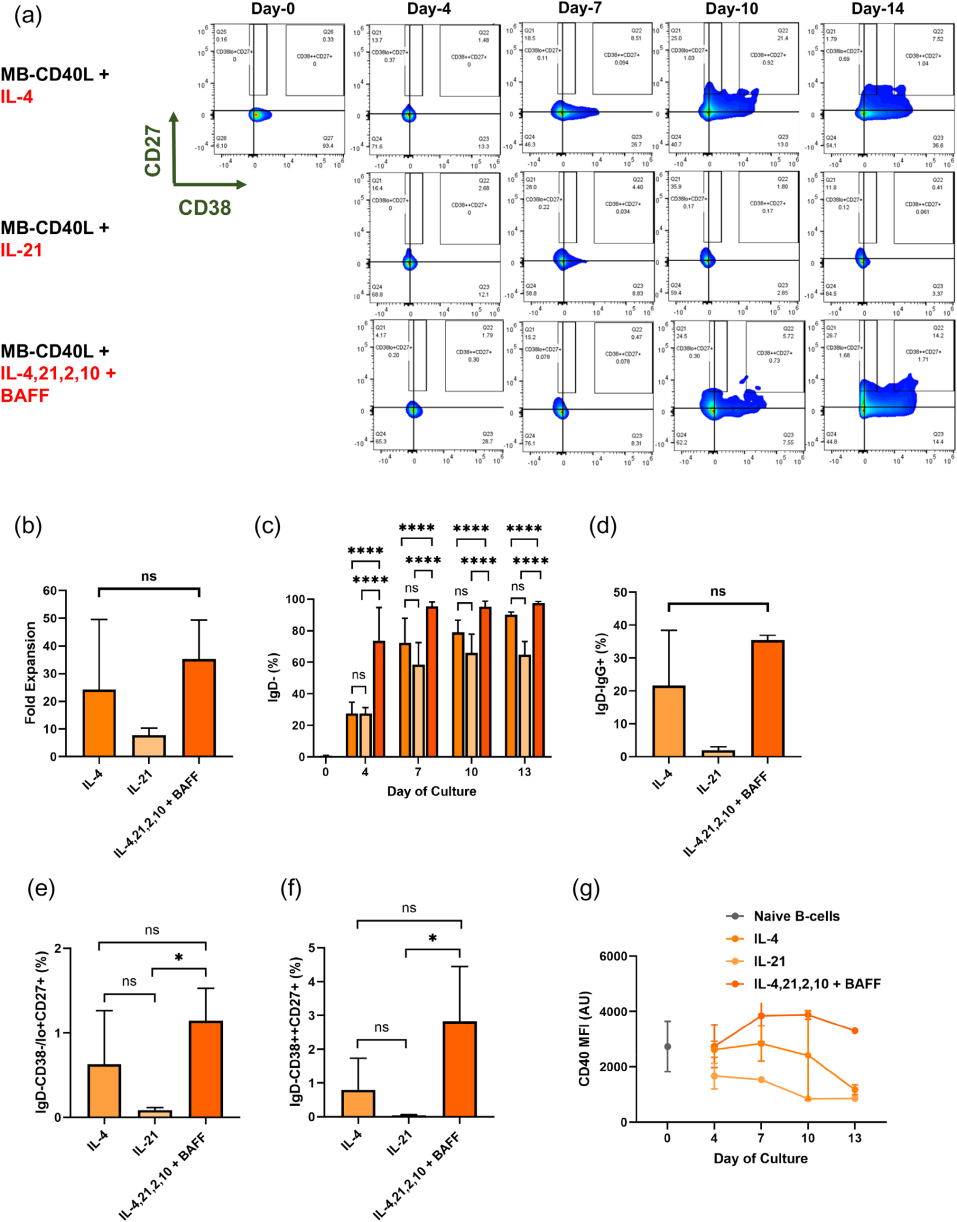
Functions of IL‐4 or IL‐21 signaling alone or together with IL‐2 and IL‐10 on the differentiation of naïve B‐cells into effector phenotypes. (a) Representative flow cytometry plots of CD27 versus CD38 for days 0, 4, 7, 10, and 14 of cultures of high‐purity naïve B‐cells activated by MB‐CD40L in the presence of indicated interleukin(s) or together with BAFF. Bar graphs for naïve B‐cell activation via MB‐CD40L with IL‐4 alone (medium orange), IL‐21 alone (light orange) or BAFF and ILs‐2, 4, 10, 21 (dark orange) comparing (b) fold expansion of B‐cells on day‐13 (c) the percentage of IgD‐ B‐cells from days 0 to 13 (d) the percentage of IgD‐IgG+ cells on day‐13, and the yield of (e) memory B‐cells and (f) antibody‐secreting cells (ASCs). (g) Median fluorescence intensity (MFI) of CD40 on activated B‐cells from days 0 to 13 for MB‐CD40L activation in the presence of IL‐4 only (medium orange), IL‐21 only (light orange) or BAFF and ILs‐2,4,10,21 (dark orange). Data were acquired as duplicates for two independent, healthy donors and presented as mean + standard deviation. Statistical analysis was conducted by applying ordinary one‐way ANOVA with Tukey's multiple comparisons (*p ≤ 0.05; ns *p* > 0.05).

As seen in the flow cytometry plots in Figure 3a, for all tested groups, CD38 was downregulated by initial activation, resulting in >80% CD38‐CD27‐ cells by day‐4. However, beyond day‐7, no further changes were observed with IL‐21 alone, whereas significant re‐expression and upregulation of CD38 were observed with IL‐4 only or with all interleukins.

Despite the negligible changes in the expression of CD38 and CD27 in the presence of IL‐21 only, it was sufficient to yield approximately 10‐fold B‐cell expansion (Figure 3b). Compared to that, MB‐CD40L activation in the presence of IL‐4 only or all interleukins yielded approximately 20‐ and 30‐fold expansion, respectively. These big differences in B‐cell expansion were, however, statistically insignificant due to the great donor variability.

More stark differences were observed in the kinetics of isotype class switching (Figure 3c, d). Starting from 100% IgD+ naïve B‐cells, at least 70% of the cells lost the expression of IgD− by day‐4 in the presence of all interleukins, whereas this yield was limited to ~25% with either IL‐4 only or IL‐21 only, indicating much slower onset of CSR (*p* < 0.0001). While continued activation via MB‐CD40L progressively increased the yield of IgD− B‐cells for all groups, significant differences were observed in both the magnitude and kinetics of IgD down‐regulation. In general, the slowest switching kinetics were observed for CD40L activation with IL‐21 only, where even after 2 weeks of activation, approximately 40% of the B‐cells still expressed IgD, followed by IL‐4 only group, where this value was approximately 30%. In comparison, all interleukins exhibited dramatically faster CSR kinetics, where almost 95% of B‐cells were IgD− as early as day‐7 (Figure 3c). While IL‐21 only condition yielded a minimal CSR to IgG after 2 weeks of culture, both the IL‐4‐only group and all‐interleukin group yielded significantly higher IgG+ populations (Figure 3D).

Finally, we measured the yield of memory B (IgD–CD38−/lo + CD27+) and ASCs (IgD–CD38++CD27+) phenotypes after 2 weeks of activation for these groups. The MB‐CD40L with only the IL‐21 group resulted in a sub‐quantifiable yield of the effector B‐cell phenotypes. Compared to this, at least mediocre increases in the expression of both CD38 and CD27 were induced by MB‐CD40L activation in the presence of IL‐4 only (Figure 3a), and the highest yields of both memory B‐cells (Figure 3e) and ASCs (Figure 3f) were achieved with all interleukins and BAFF among the tested groups.

Many downstream signaling cascades of each interleukin can cause these differences in B‐cell differentiation. As a synergistic mechanism, we found that the expression levels of CD40 on activated B‐cells in each group are clearly different. As seen in Figure 3g, CD40 was downregulated by IL‐21 only as early as day‐4, and this downregulation continued throughout 2‐week culture. Therefore, with IL‐21 only, signaling provided MB‐CD40L would be minimal. In contrast, in the presence of all interleukins, an increase in the expression of CD40 was observed at least until day‐7, before it is downregulated in the second week of activation. With IL‐4 only, CD40 expression was similar to naïve B‐cells (day 0) until day‐7 and was downregulated in the second week.

In summary, MB‐CD40L activation with IL‐21 alone is insufficient to differentiate naïve B‐cell to effector B‐cell phenotypes, although it can expand B‐cells to a certain degree. The presence of IL‐4 is critical for further differentiation of B‐cells after initial activation, and the addition of the other interleukins is synergistic for the significant improvement in the yield of memory B‐cell and ASC phenotypes.

### 2.3 Comparison of feeder‐free CD40 activation methods

**FIGURE 4:**
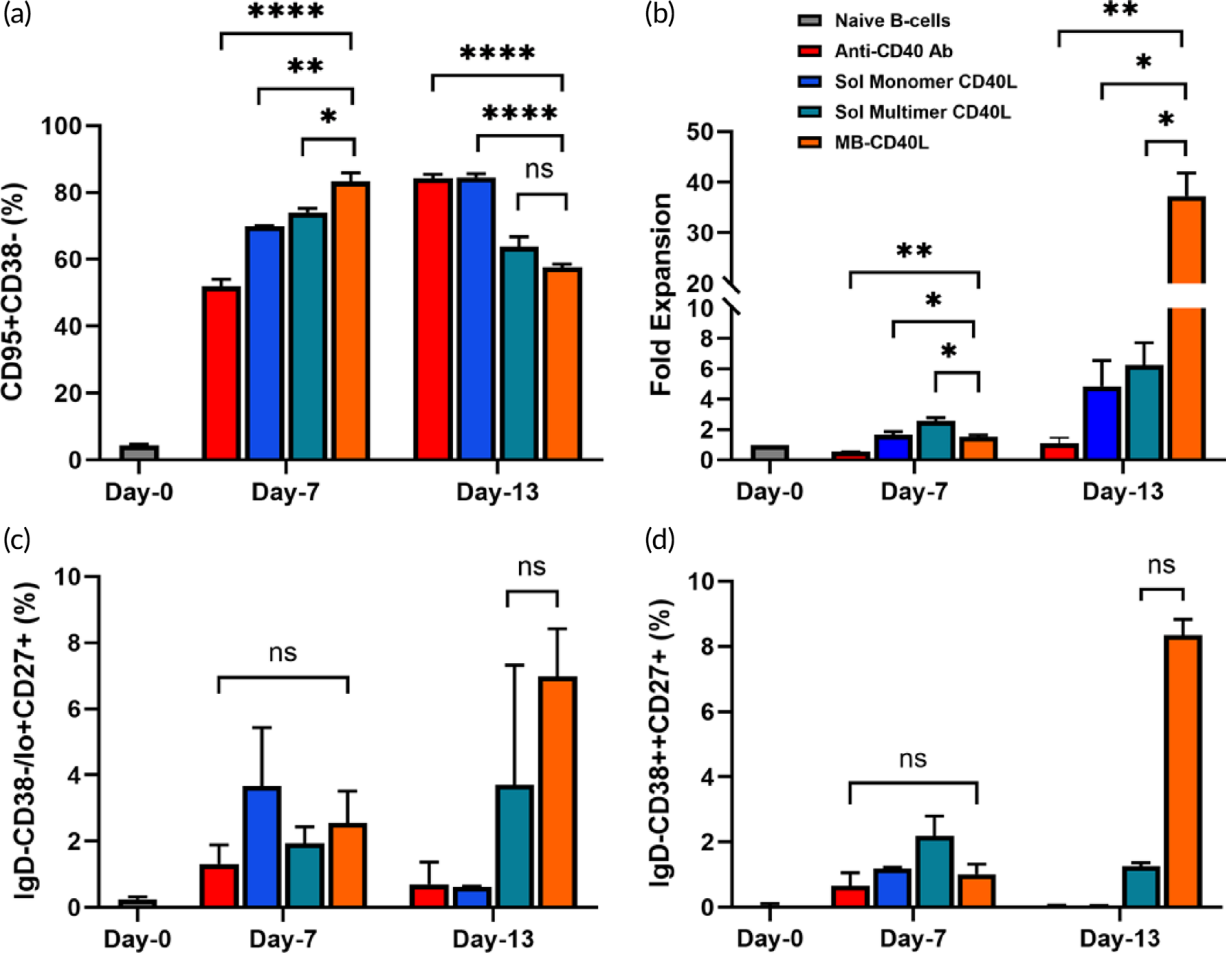
Comparison of feeder‐free CD40 activation methods on expansion and differentiation of human naïve B‐cells. Bar graphs comparing the yield of (a) GC B‐cells, (b) fold expansion in number of total B‐cells, (c) memory B‐cell yield, and (d) ASC yield on days‐7 and 13 for naïve B‐cells (gray) activated with soluble monomeric CD40L (blue), anti‐human CD40 antibody (red), soluble multimeric CD40L (dark cyan) and MB‐CD40L (orange). Data were acquired each day as duplicates for each group and presented as mean + standard deviation. Statistical analysis was conducted by applying two‐way ANOVA with Tukey's multiple comparisons (*p ≤ 0.05; **p ≤ 0.01; ****p ≤ 0.0001; ns *p* > 0.05).

Based on the microscope imaging, we showed that MB‐CD40L was more efficient in the early activation of B‐cells compared to soluble monomeric or multimeric CD40L presentation in the presence of IL‐4 and BAFF (Figure 1). We further examined the efficiency of different feeder‐free CD40L presentations in B‐cell expansion and differentiation with the optimized soluble factors, namely IL‐4, ‐2, ‐10, ‐21, and BAFF. Here, we compared MB‐CD40L with monomeric and multimeric soluble CD40L as well as soluble agonistic anti‐CD40 antibody. While the CD40L dosage was maintained at 100 ng per mL of culture medium for all CD40L presentation groups, the anti‐CD40 antibody was added at a final concentration of 1 μg/mL in accordance with prior studies.^21^, ^22^ The anti‐CD40 antibody was evidently the least efficient in inducing GC B‐cells, while MB‐CD40L was the most efficient by day‐7 (Figure 4a). While anti‐CD40 antibody and monomeric CD40L continued to increase the GC B‐cells by day‐13, the multimeric CD40L and MB‐CD40L groups further differentiated the GC B‐cells into other effector phenotypes (Figure 4c, d). The yields of memory B‐cells (Figure 4c) and ASCs (Figure 4d) were indifferent for all groups by day‐7 (*p* > 0.05), but at the end of the second week (day‐13), multimeric soluble CD40L and MB‐CD40L yielded significantly higher percentages of both effector phenotypes than the other two.

Meanwhile, B‐cell expansion was slow for all groups, resulting in less than 2‐fold improvement by day‐7, with the soluble multimeric CD40L group producing the highest yield. However, dramatic changes in B‐cell expansion were observed in the second week. While the anti‐CD40 antibody group could not produce any appreciable expansion, the soluble monomeric and multimeric CD40L presentation yielded ~5‐fold expansion. In comparison, the MB‐CD40L group yielded as much as 40‐fold expansion.

### 2.4 Synergistic effects of CD40L, BCR, and TLR signaling on the expansion and differentiation of human naïve B‐cells

To improve the yield of effector B‐cells from naïve B‐cells, we explored the addition of other signaling factors that would complement the activation by MB‐CD40L. To this end, we pursued the activation of the BCR by using antibodies targeting the immunoglobulin receptors, namely, IgM, IgG, and IgA, the CD21 co‐receptor, and the toll‐like receptor 9 (TLR‐9) by using the TLR‐9 agonist, a synthetic CpG ODN.^32^

**FIGURE 5:**
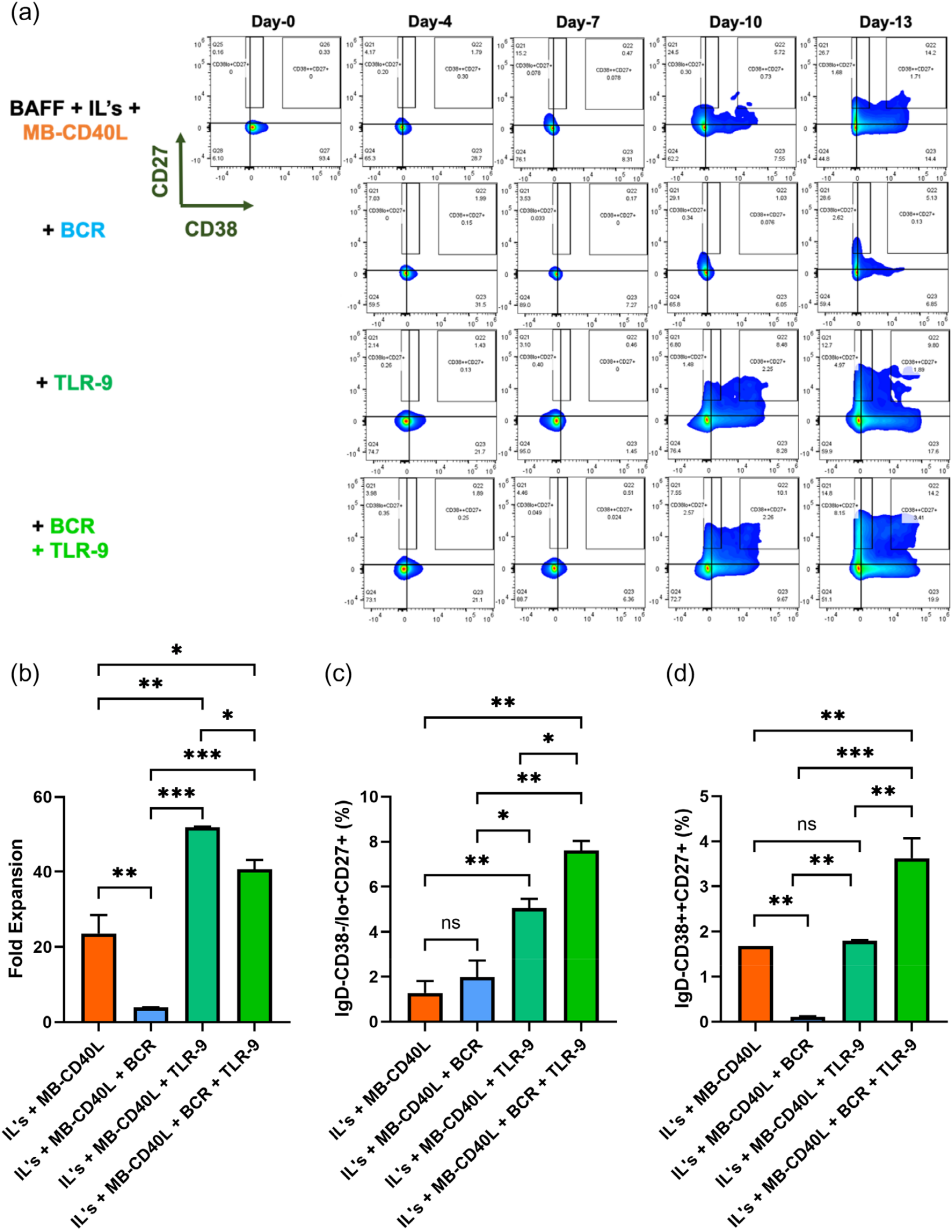
Synergistic activation by MB‐CD40L, BCR, and TLR‐9 signaling for improved B‐cell expansion and differentiation. (a) Representative flow cytometry plot of CD27 versus CD38 for days 0–13 cells (gated on IgD‐ B‐cells from day‐4 onwards) for the activation of naïve B‐cells by MB‐CD40L with or without the B‐cell receptor signal by anti‐human IgM/G/A (H + L) antibody plus co‐receptor signal by anti‐human CD21 antibody (referred to as BCR) and/or toll‐like receptor signal by the TLR‐9 agonist CpG ODN 2006 in the presence of BAFF and the interleukins Il‐2, ‐4, ‐10 and ‐21. Bar graphs of (b) fold expansion and the yield of (c) memory B‐cells and (d) ASCs on day‐13 for each group. Data were acquired each day as duplicates for each group and represented as mean + standard deviation. Statistical analysis was conducted by applying ordinary one‐way ANOVA with Tukey's multiple comparisons (*p < 0.05; **p ≤ 0.01; ***p ≤ 0.001; ns *p* > 0.05).

Representative flow cytometry plots in Figure 5a show that irrespective of the activation factors in culture, negligible differences in CD38 and CD27 expression were observed between the groups in week‐1 (up to day‐7). As discussed above, the MB‐CD40L alone induced an increase in CD38 and a minimal increase in CD27 expression in the presence of interleukins and BAFF by day‐10. The addition of BCR and CD21 co‐receptor activations, however, reduced the expression of both CD27 and CD38. In comparison, those groups with additional TLR‐9 signaling with or without BCR and CD21 co‐receptor activations showed distinct increases in CD38 and CD27 expression by day‐10. The group with BCR and co‐receptor signaling in the presence of TLR‐9 agonist was the most effective in the induction of ASC phenotypes (CD27 + CD38++) by day‐13.

Figure 5b shows the fold expansion of B‐cell numbers for each condition. The addition of BCR and co‐receptor signaling not only prohibited the differentiation of naïve B‐cells but also significantly reduced the overall expansion of B‐cells, from ~20‐fold expansion with MB‐CD40L only condition to less than 5‐fold in the presence of BCR and co‐receptor signaling. In stark contrast, co‐activation with TLR‐9 agonist together with MB‐CD40L exceeded the fold expansion observed with CD40L activation alone by >2×, resulting in 50‐fold net expansion by day‐13. The competing effects of BCR/co‐receptor and TLR‐9 signaling on B‐cell expansion were observed in the condition with all three signaling factors, MB‐CD40L, BCR and co‐receptor, and TLR‐9, which yielded ~40‐fold expansion.

Overall, the yields of memory B‐cell and ASC phenotypes by day‐13 of culture are summarized in Figure 5c, d, respectively. The additional activation of the BCR and the co‐receptor induced no significant change in the yield of memory B‐cells while significantly reducing the yield of ASCs. In comparison, the co‐activation of TLR‐9 with CD40 significantly improved the yield of memory B‐cells but not ASCs. In synergy with CD40 and TLR‐9, however, the addition of BCR and co‐receptor activation further increased the yields of both memory B‐cells and ASCs.

Altogether, the activation of BCR and co‐receptor negates further differentiation of GC B‐cells and negatively regulates the expansion induced by early activation of MB‐CD40L alone. However, a positive synergy can happen in the presence of both CD40 and TLR‐9 activation signaling, which improves the overall yields of both memory B‐cells and ASCs.

### 2.5 Further optimization of synthetic niche using modular design—temporal regulation of IL‐4 and IL‐21 as well as quantity of CD40L signaling

The in vitro culture conditions presented in this report are feeder‐ and serum‐free conditions specifically designed for the manufacturing of therapeutic B‐cells in mind. This synthetic and modular nature of the artificial niches also enables precise control over the quantity and quality of signaling factors and temporal regulation. While testing every permutation of these conditions is not feasible in a single paper, we wanted to demonstrate this unique aspect of the synthetic niche by changing some critical soluble factors and the CD40L dosage temporally. Previously, it was shown that (i) IL‐4 is the primary factor required for CD19+ B‐cell differentiation into the memory B‐cell phenotype, (ii) the differentiation of memory B‐cells (CD38‐/lo + CD27+) to ASCs (CD38++CD27+) is induced only by IL‐21, and (iii) IL‐4 has an inhibitory role on the IL‐21‐induced terminal differentiation into ASCs.^38^ Additionally, while CD40 signaling is necessary for the survival of the differentiated B‐cells in culture, a high dosage of CD40L has been shown to have a negative effect on the terminal differentiation into plasma cells or ASCs.^15^, ^31^

Here, we tested if these previous findings are valid for the starting population strictly limited to human naïve B‐cells from multiple donors. We activated the naïve B‐cells with MB‐CD40L, BCR and co‐receptor, and TLR‐9 signaling factors in the presence of all IL‐2, IL‐4, IL‐10, and IL‐21 and BAFF up to day‐10. On day‐10, when the activated B‐cells express CD27, we removed either IL‐21 only or BCR and IL‐4 from the culture, while all other factors were maintained. Furthermore, we also explored the effect of reduced CD40L dosage during this terminal differentiation phase (between day‐10 and day‐13). To reduce the CD40L dosage by 10‐fold (which we refer to as MB‐CD40L^low^), we lowered the surface density of CD40L on the microbeads while maintaining the cell‐to‐bead ratio constant.

**FIGURE 6:**
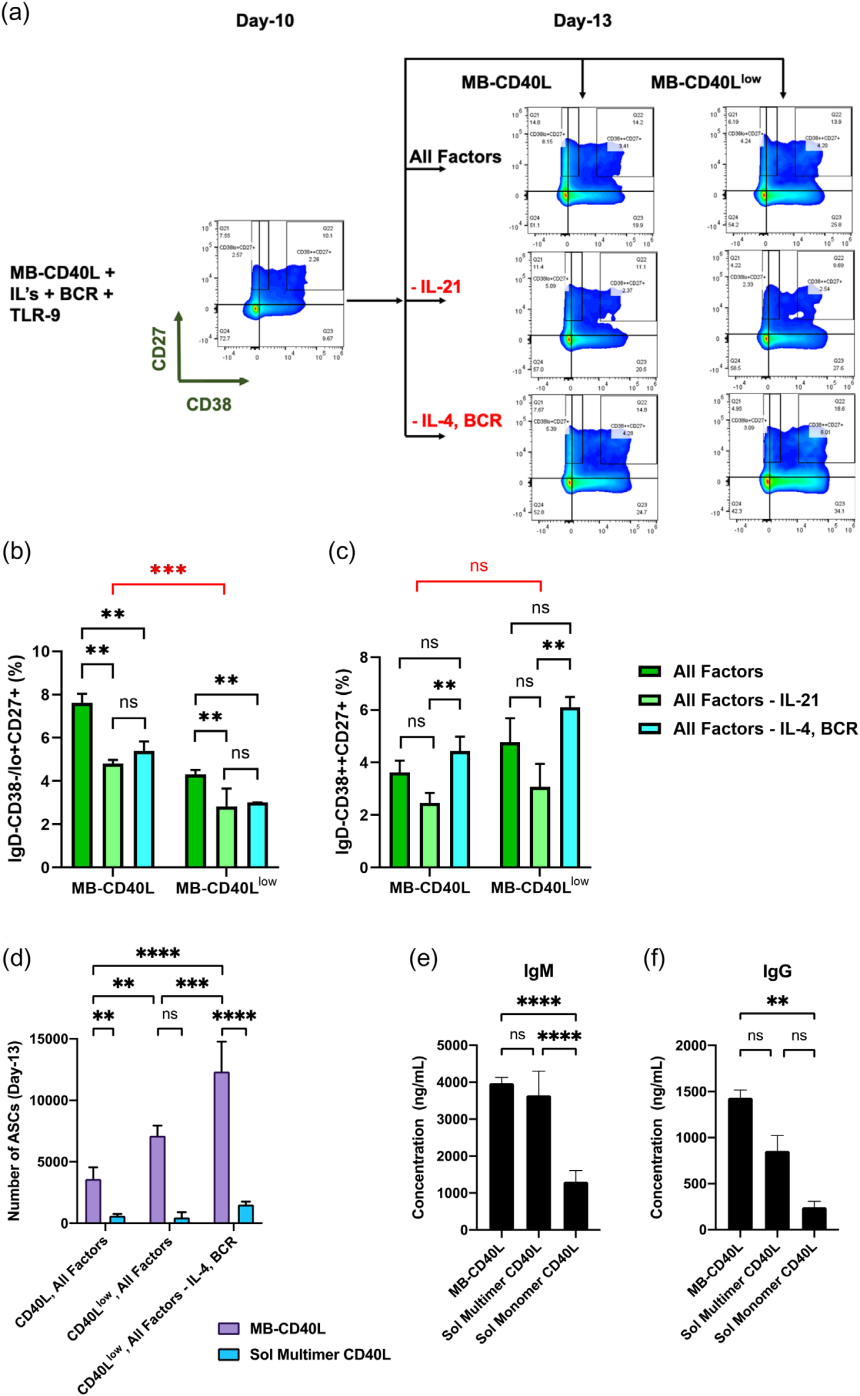
Switching interleukins and CD40L dosage for the terminal differentiation of naïve B‐cells for effective generation of functional ASCs. (a) Representative flow cytometry plot of CD27 versus CD38 gated on IgD‐ B‐cells on day‐10 and day‐13. High‐purity naïve B‐cells were activated with MB‐CD40L, BAFF, anti‐BCR/CD21 antibodies, TLR‐9 agonist, and IL‐2, IL‐4, IL‐10, and IL‐21 for initial 10 days. On day‐10, CD40L dosage was either maintained (MB‐CD40L) or reduced by 10‐fold (MB‐CD40L^low^). For each CD40L dosage, three different conditions were tested up to day‐13: All factors or all factors except IL‐21 or all factors except anti‐BCR antibodies and IL‐4. (b), (c) Bar graphs comparing the yield percentage of memory B‐cells (IgD‐CD38‐/lo + CD27) and ASCs (IgD‐CD38++CD27+) on day‐13 from indicated conditions. Data were acquired as duplicates and presented as mean + standard deviation. (d) Number of ASCs generated by day‐13 from the culture of 10,000 naïve B‐cells (day‐0). Either MB‐CD40L or Sol Multimer CD40L was employed for the entirety of the culture period. Other indicated culture conditions were varied between day‐10 and day‐13. Data were acquired as triplicates and presented as mean + standard deviation. (e), (f) The concentrations of antibodies of different isotypes, IgM and IgG, secreted by ASCs generated by activation of high‐purity naïve B‐cells using the indicated method of CD40L presentation in the presence of BAFF, anti‐BCR antibodies, TLR‐9 agonist, and IL‐2, IL‐4, IL‐10, and IL‐21 for the initial 10 days. On day‐10, cells were reseeded at 10,000 cells per well, CD40L dosage was reduced by 10‐fold, and all soluble factors except anti‐BCR antibodies and IL‐4 were employed. ELISA was performed using supernatant samples harvested on day‐12. Data were acquired as triplicates and presented as mean + standard deviation. (b)−(f) Statistical analysis was conducted by applying two‐way ANOVA with Tukey's multiple comparisons (*p < 0.05; **p ≤ 0.01; ***p ≤ 0.001, ****p ≤ 0.0001; ns *p* > 0.05).

Figure 6a shows the effects of the modifications in culture conditions between day‐10 and day‐13 with the representative flow cytometry plots of CD27 versus CD38. Overall, lowering the CD40L dosage on day‐10 reduced the yield of memory B‐cells (Figure 6b). Thus, maintaining all employed soluble factors together with the original CD40L dosage resulted in the highest yield of memory B‐cells (Figure 6b). While lowering the CD40L dosage on day‐10 only mildly increased (statistically insignificant) the yield of ASCs (Figure 6c), removing BCR/co‐receptor signaling and IL‐4 on day‐10 significantly improved the yield of ASCs (Figure 6c).

Overall, based on these results, we identified the best culture conditions for the highest yields of memory B‐cells or ASCs, respectively. First, maintaining MB‐CD40L together with BCR and TLR‐9 signals, all tested interleukins, and BAFF up to day‐13 favors the generation of memory B‐cells. Second, lowering the CD40L dosage by changing it to MB‐CD40L^low^ and simultaneous removal of IL‐4 and BCR signaling from the culture during the terminal differentiation phase (i.e., from day‐10 to day‐13) favors the generation of ASCs. When we conducted experiments on the naïve B‐cells from four different donors, despite small donor variability in the absolute yields, the reported culture‐condition‐dependent differentiation shift was reproducibly observed (Supplementary Figure 2).

We further compared the performances of MB‐CD40L and the soluble multimeric form of CD40L with the identified culture condition optimized for generating ASCs. MB‐CD40L generated a significantly higher number of cells with ASC phenotype than the soluble multimer (Figure 6d). In order to test whether the ASCs generated within the synthetic culture niches are indeed functional, that is, secret a significant amount of antibodies, we measured the levels of IgM (Figure 6e) and IgG (Figure 6f) secreted by the normalized number of activated B‐cells (10,000 cells seeded on day‐10) by ELISA. We confirmed that not only the ASCs generated from the synthetic culture niches are functional regardless of CD40L presentation methods, but also the MB‐CD40L generates higher percentages of functional ASCs secreting IgM or IgG isotypes, compared to soluble monomer CD40L (Figure 6e, f).

### 2.6 Clonal expansion, isotype class switching, and somatic mutations during artificial germinal center (aGC) reactions

**FIGURE 7:**
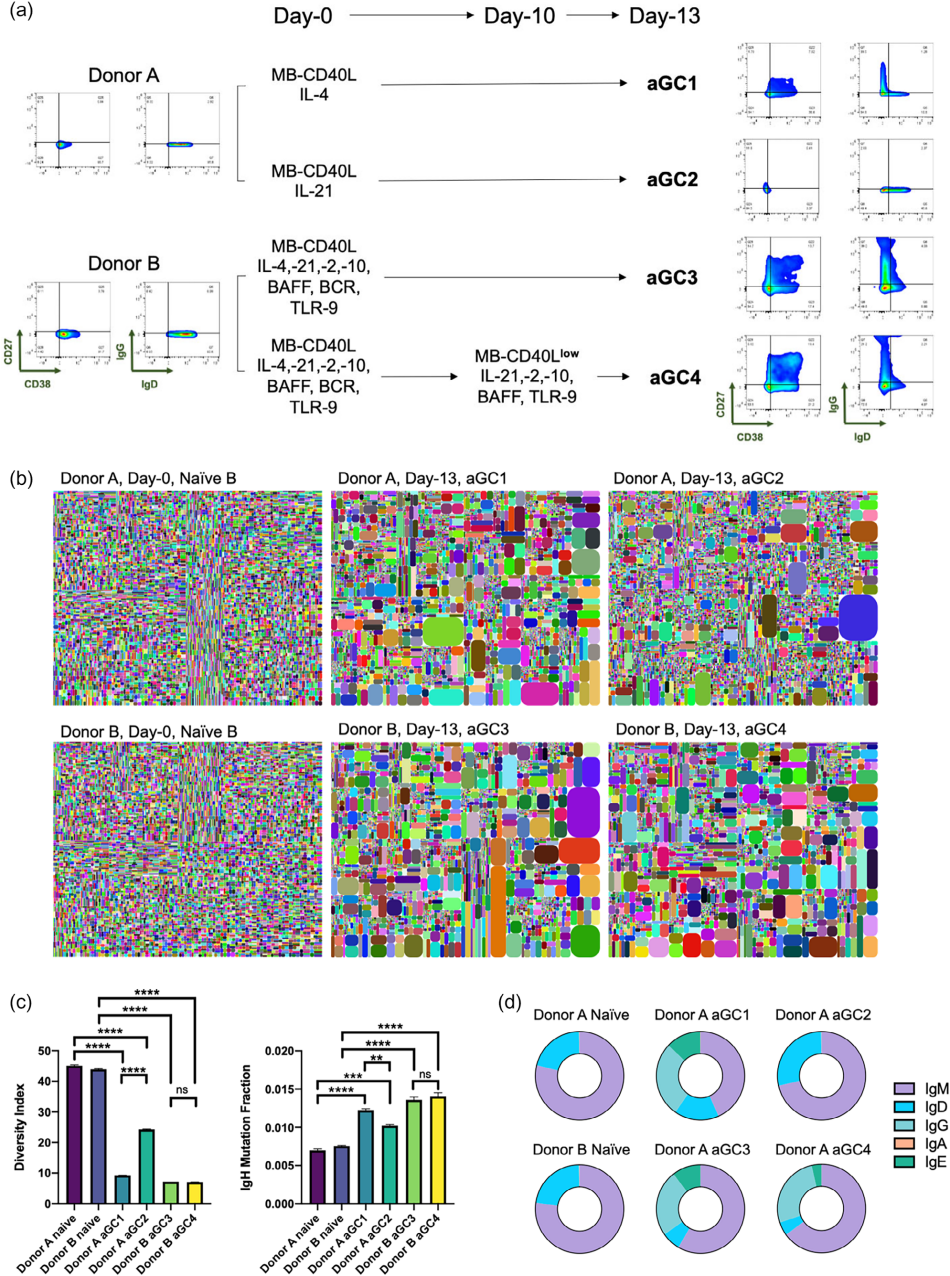
Examination of B‐cell receptor (BCR) repertoire by sequencing immunoglobulin heavy chain (IgH) gene of naïve B‐cells (day‐0) and artificial germinal center (aGC) B‐cells (day‐13). (a) Representative flow cytometry plot of CD27 versus CD38 and IgG versus IgD, before (day‐0, naïve B‐cells) and after (day‐13) four different aGC cultures with respective conditions. (b) The tree maps of two naïve B‐cell populations from two different donors, and the corresponding four different aGC B‐cells after 13‐day culture. (c) Diversity index (left) and the frequency of mutations within IgH of each sample, the bar graph shows mean + standard deviation from the duplicate. Statistical analysis was conducted by applying one‐way ANOVA with Tukey's multiple comparisons (**p ≤ 0.01; ***p ≤ 0.001; ****p ≤ 0.0001; ns *p* > 0.05). (d) Proportion of isotypes of each sample as a pie chart.

As shown above, the MB‐CD40L‐based feeder‐free in vitro culture conditions can induce a partial list of reactions that naïve B‐cells would go through within a physiological GC, namely rapid B‐cell expansion, class switching recombination (CSR), and differentiation to effector B‐cells. Here, we examined how BCR repertoires evolve within four different artificial GC (aGC) conditions by analyzing the Ig‐heavy chain gene sequences before and after the respective 13‐day cultures. The naïve B‐cells were initially activated with MB‐CD40L in all conditions, but we varied the soluble factors: IL‐4 only for aGC1, IL‐21 only for aGC2, or a combination of IL‐4, ‐21, ‐2, ‐10, BCR signaling factors, and BAFF for aGC3. The condition for aGC4 was identical for aGC3, except we switched MB‐CD40L to MB‐CD40L^low^ and excluded IL‐4 and BCR signaling factors from the culture on day‐10 (Figure 7a). Flow cytometry analyses confirmed that the outcomes are reproducible to the data shown above. Briefly, as shown in Figure 3, in the presence of MB‐CD40L, IL‐4 alone (aGC1) was able to induce some degree of differentiation of B‐cells to effector phenotypes and isotype class switching to IgG, while IL‐21 alone (aGC2) cannot (Figure 7a). As shown in Figure 6, additional signaling factors in aGC3 and aGC4 increased the yield of effector B‐cells and IgG+ cells, and the reduction of CD40L dosage and removal of IL‐4 and BCR signaling at the later stage of culture promoted further differentiation to ASCs (Figure 7a).

The tree maps were generated as an illustrative demonstration of the diversity of the BCR repertoire within each sample (Figure 7b, “Method” section). Large BCR repertoires are present in both naïve B‐cell populations from two different healthy donors. The relative frequencies of all BCR clones are similar, which supports that our starting B‐cell populations are truly naïve (Figure 7b). It was intriguing to observe that some degree of selective clonal expansion was observed in all aGC conditions (Figure 7b). To quantify the diversity of each BCR repertoire, we calculated the diversity index (DI, “Method” section, Supplementary Figure 3), where the higher the DI value is (up to 50), the more diverse the repertoire is. The calculated DI values were 44.9 and 44.1 for the two naïve B‐cells on day‐0, and 9.3, 24.2, 7.2, and 7.1 for aGC1 through aGC4, respectively (Figure 7c, left). It is evident that some BCR clones selectively expanded during the 13‐day aGC cultures, even in the absence of universal BCR signaling.

We also counted the frequencies of all mutations in IgV sequences to evaluate if SHM is induced in the aGC conditions (Figure 7C, right). The mutation frequencies observed on B‐cells in all aGC conditions were slightly higher than on naïve B‐cells. However, the highest frequency observed from aGC 4 (0.0141 ± 0.00046) was a mere 1.93 times bigger than the average frequency found in naïve B‐cells (0.0073 ± 0.00018). In comparison, the pan B‐cells isolated from PBMCs of a healthy donor showed a mutation frequency of 0.0416 ± 0.00042, which is 5.7 times bigger than the naïve B‐cells.

Lastly, we examined the isotype classes of all heavy chain sequences and calculated the frequency of each isotype (Figure 7d, Supplementary Figure 4). The naïve B‐cells from both donors show more than 99.5% of sequences with non‐switched isotypes, that is, IgM or IgD. Like other GC reactions, the aGC2 condition with IL‐21 alone as the soluble factor did not successfully induce CSR. In stark comparison, significant CSRs to IgG and IgE were induced in all the other three aGC conditions (Figure 7d), and high frequencies of BCR clones were detected with 3 or more kinds of isotype classes, which strongly support that true CSRs were induced during the aGC cultures.

## 3 DISCUSSION

In this paper, we explored serum‐free, feeder cell‐free culture conditions for effective expansion and differentiation of human naïve B‐cells into memory B‐cells and ASCs. In physiology, such activation happens within the specialized microenvironment called GC in secondary lymphoid organs, which results in clonal expansion, CSR of immunoglobulin receptors, and affinity maturation via multiple rounds of SHMs along with terminal differentiation into effector B‐cells. For the GC reactions, various molecular signals provided by follicular helper T (T_FH_) cells are critical.^39^ We tried to recapitulate the CD40L presented by the T_FH_ cell membrane by designing artificial T_FH_ cells using commercially available streptavidin‐labeled iron oxide magnetic microbeads (MBs) that have been widely used in the manufacturing of immune cell therapeutics. While a similar trial has been previously reported for activation of murine B‐cells,^27^ to the best of our knowledge, this is the first report to apply MB‐CD40L activation on primary human B‐cells.

First, we demonstrated that MB‐CD40L can effectively interact with human naïve B‐cells to induce better initial activation (Figure 1) and induce higher expansion and differentiation to effector B‐cells (Figures 4 and 6), compared to soluble counterparts. The enhanced activation potency may be attributed to multiple aspects, including a higher valency of CD40L on the surface, mechanical transduction of the CD40 signaling, and the three‐dimensional nature of MB‐based presentation of CD40L ligands. It has been previously shown that the magnitude of B‐cell proliferation by CD40L depends upon its degree of oligomerization.^40^ It was also shown that the membrane‐bound recombinant CD40L has a much lower signaling threshold compared to soluble antibodies.^21^, ^22^

Another critical aspect of this paper that is worth noting is that we also established a standardized B‐cell activation protocol that reproducibly yields expected B‐cell phenotypes for potential use in the manufacturing of B‐cell therapeutics. We achieved this by restricting our starting population to naïve B‐cells with >95% purity instead of all CD19+ B‐cells from PBMCs. In particular, we minimized the contamination of double‐negative (DN) B‐cells by employing two‐step MACS isolation. The DN B‐cells are a type of pre‐activated B‐cells that exhibit higher levels of SHM in comparison to the naïve B‐cells, confirming their GC origin, express IgG and IgA, and have the potential to differentiate into plasma cells via extrafollicular reactions (i.e., outside of GC).^41^, ^42^, ^43^ The presence of a small population of DN B‐cells within the starting B‐cell population on day‐0 not only increases the kinetics of differentiation but also yields much higher memory B‐cells and ASCs,^32^, ^33^, ^36^, ^37^, ^38^, ^44^ which, ironically, is responsible for unreliable and unpredictable culture outcomes and significant lab‐to‐lab and donor‐to‐donor variabilities. Thus, we eliminated the DN B‐cells from our starting population, which enabled our culture protocol to be standardized, yield reproducible outcomes over multiple donors (Supplementary Figure 2), and better recapitulate the physiological activation of naïve B‐cells within the GC.

Using the synthetic and modular nature of the feeder‐free culture condition using MB‐CD40L, we systematically tested the effects of various soluble factors in the activation of naïve B‐cells. As IL‐4 and IL‐21 have been known as critical factors for GC reactions,^38^, ^45^, ^46^, ^47^, ^48^, ^49^ we tested them first. Interestingly, while IL‐4 alone as a soluble factor induced a mediocre expansion, differentiation, and CSR, IL‐21 alone could not (Figures 3 and 7). In the presence of IL‐4, however, the additional soluble factors, namely IL‐2, IL‐10, IL‐21, and BAFF, enhanced the GC reactions induced by MB‐CD40L. We showed that this enhancement of GC reactions is at least partially due to the upregulated expression of CD40 receptors with the combination of soluble factors (Figure 3).

To improve the efficiency of overall aGC reactions, we tried to complement T‐dependent activation by MB‐CD40L with T‐independent activation mechanisms using soluble reagents that can activate BCR and CD21 co‐receptor as well as the TLR‐9 receptor. However, the addition of BCR and co‐receptor signals alone negatively impacted the overall expansion and differentiation of naïve B‐cells (Figure 5). In comparison, the addition of the TLR‐9 signaling alone enhanced the expansion and differentiation of naïve‐B‐cells to memory‐B‐cells, and it makes the BCR and co‐receptor signals synergistically contribute to the GC reactions as well (Figure 5), which agrees well with previous reports.^20^, ^29^, ^32^

In our culture, the successful differentiation of the naïve B‐cells first induced the emergence of memory B‐cell phenotype by day‐7 and the population continued to grow, while the ASC phenotype emerged later around day‐10 and grew. Thus, we divided the culture into two phases and varied the culture conditions. In the first phase up to day‐10, we tried to maximize the differentiation to memory B‐cells by combining T‐dependent and T‐independent signals with all the soluble factors. Then we selectively varied the conditions for the final outcomes to incline to either memory B‐cells or ASCs. Based on the previous literature, we applied these rationales: (i) IL‐21 plays a significant role in B‐cell differentiation toward plasma cells, (ii) IL‐4 inhibits the IL‐21‐mediated differentiation to ASCs,^31^, ^38^ (iii) IL‐2, IL‐10, and BAFF can enhance both early and late differentiation phases,^50^ (iv) low CD40L signaling of the memory B‐cells enhances differentiation toward ASCs,^31^ and (v) BCR signaling is not needed after the terminal differentiation of memory B‐cells to ASCs. Indeed, when the regular dosage of CD40L together with BCR and TLR9 signals in the presence of all the soluble factors were maintained, a maximum yield of memory B‐cells was achieved. Meanwhile, lowering the dosage of CD40L by one magnitude and the simultaneous removal of BCR factors and IL‐4 on day‐10 induced a maximized yield of ASCs on day‐13. The functionality of the ASCs generated by the aGC culture conditions was confirmed by measuring the concentration of secreted antibodies (IgM and IgG) using ELISA (Figure 6).

Results from sequencing of immunoglobulin heavy chain gene of the differentiated B‐cells from our culture were intriguing. First, regardless of the universal application of BCR and co‐receptor signaling, there was some selective expansion of certain clones (Figure 7). The sequencing results also agree well with the flow cytometry data showing that GC reactions such as robust expansion and CSR can be successfully initiated by IL‐4 alone, but not by IL‐21 alone, in the presence of MB‐CD40L. Interestingly, the frequencies of mutations within IgH of the differentiated B‐cells from all aGC cultures were slightly higher than the naïve B‐cells, even if this increase was relatively mediocre compared to the frequencies observed in the B‐cells from physiological GCs. The low level or lack of SHM induction agrees with other previous studies on in vitro activation of naïve B‐cells.^20^, ^29^, ^32^, ^44^ The successful induction of CSR to IgG+ B‐cells proves the potential of aGC culture for generating effector B‐cell therapeutics with isotype‐specific humoral immunity. It is worth noting that although the differentiation to IgG+ B‐cells is of primary interest in vaccine development, by virtue of its higher valency, IgM complements the IgG potency in both healthy and immune‐deficient individuals, and recent studies showed that it can be even more potent than IgG antibodies in neutralizing multiple variants of SARS‐CoV‐2,^51^, ^52^ which warrants further investigation on IgM‐secreting B‐cell therapeutics.

## 4 CONCLUSIONS

An effective in vitro culture system capable of activating human B‐cells from therapeutically relevant sources, such as peripheral blood, is urgently needed to advance B‐cell therapies. To overcome the limitations of current protocols that use feeder cells and serums, we present a microbead‐based synthetic niche that effectively converts human naïve B‐cells into memory B and ASC phenotypes, accompanied by isotype switching and significant expansion. This robust, feeder‐free, and serum‐free culture system holds promise for the manufacturing of B‐cell therapeutics.

## 5 MATERIALS AND METHODS

### 5.1 Isolation of naïve B‐cells from frozen human PBMC's via two‐step MACS

Human naïve B‐cells were isolated from frozen PBMC's (70025.1; Stemcell Technologies, Canada) using a commercially available MACS isolation kit (130091150; Miltenyi Biotec, USA) following manufacturer‐recommended protocol. Briefly, for one‐step isolation, the PBMCs were incubated with the Naïve B‐cell Antibody Cocktail and Anti‐Biotin Microbeads before being passed through the MS column (130042201; Miltenyi Biotec, USA), which resulted in approximately ~92% purity naïve B‐cells (IgD + CD27‐) plus the DN B‐cells (IgD‐CD27‐). To remove the DN B‐cells, we further incubated the isolated B‐cells from the first step with biotinylated anti‐human IgG (130119877; Miltenyi Biotec, USA) and biotinylated anti‐human IgA (130113474; Miltenyi Biotec, USA) antibodies, and then passed through the MS column after incubating with the Anti‐Biotin Microbeads in the kit, which reproducibly yielded a high‐purity (>95%) naïve B‐cells.

### 5.2 Preparation of soluble multimer complex of recombinant CD40L

In this study, we used a recombinant CD40 ligand (Gly 116—Leu 261, CDL‐H82Db‐25ug; Acrobiosystems, USA) in an active trimer form that is enzymatically biotinylated on its N‐terminal Avi tag, which we will designate as soluble “monomer,” for this trimeric form is a single functional molecular unit. In order to prepare the soluble “multimer” complex form of the CD40L, the biotinylated CD40L monomer was incubated with streptavidin (Thermo Scientific™ 21122; Invitrogen, USA) at a molar ratio of 1:1 for 1.5 h at room temperature. This mixture was added to the respective culture medium at a final CD40L concentration of 100 ng/mL without further purification.

### 5.3 Preparation of CD40L‐presenting microbeads

Typically, 10 μL of iron oxide microbeads suspension (10 mg/mL, 11205D; Dynabeads™ M‐280 Streptavidin, Invitrogen, USA) was washed using 1 mL of the buffer containing 1× PBS, 0.5% BSA and 2 mM EDTA via magnetic precipitation on the DynaMag stand (12321D; DynaMags™—2 Magnet, Invitrogen, USA) and resuspended in 100 μL of the same buffer. To prepare microbead CD40L (MB‐CD40L), 5 μL stock solution of biotinylated recombinant human CD40L (200 ng/ μL) was added to the microbeads suspension for MB‐CD40L^high^, vortexed briefly, and then incubated for 1.5 h on a rotary shaker. Similarly, to prepare the low surface density microbead CD40L (MB‐CD40L^low^), 2 μL stock solution of the biotinylated recombinant human CD40L (200 ng/μL) was diluted 10× using the buffer, and then 5 μL from the diluted stock was added to the microbeads suspension. After incubation, 1 mL of buffer was added, and the microbeads were washed again via magnetic precipitation to remove unbound CD40L.

### 5.4 In vitro B‐cell culture

Naïve B‐cells isolated via one‐ or two‐step MACS were seeded at a seeding density of 1 × 10^5^ cells per mL in 100 μL of the culture medium per well in 96‐well culture plates (Corning™ 3596; Corning, USA) on day‐0. A serum‐free culture media^53^ consisting of Iscove's Modified Dulbecco Medium (IMDM) (12440053; Gibco, USA) substituted with 20% BIT (9500; Stemcell Technologies, Canada) (containing 10 mg/mL bovine serum albumin, 10 μg/mL recombinant human insulin and 200 μg/mL human transferrin), 20 μg/mL low‐density lipoprotein (LDL) (L8292; Millipore Sigma, USA) and 50 μM beta‐mercaptoethanol (21985023; Gibco, USA) was used in our study. This serum‐free medium was supplemented with recombinant human BAFF, IL‐4, IL‐2, IL‐10, and IL‐21 (all from Peprotech, USA), at 10 ng/mL. Unless otherwise specified, CD40L‐presenting iron oxide microbeads were added at the specified dosages of CD40L (100 ng/mL or 10 ng/mL) at a cell‐to‐bead ratio of 1‐to‐7. For the activation of the B‐cell receptor, a combination of goat anti‐human IgA + IgG + IgM (H + L) antibody (109006064; Jackson Immuno Research Laboratories, USA) was added to the culture at 6 μg/mL concentration, together with a monoclonal mouse anti‐human CD21 antibody (MAB4909; R&D Systems, USA) at 1 μg/mL. For TLR activation, human TLR‐9 agonist ODN2006 (tlrl‐2006; InvivoGen, USA) was added to the culture medium at 2 μM concentration. In a typical experiment, cells were reseeded to 1 × 10^5^ cells per mL on day‐4 and day‐10; On day‐7, 100 μL of fresh medium containing all specified factors was added to each well without reseeding. For those experiments not involving the TLR activation, each well was topped up with 100 μL of fresh media containing the respective factors on day‐4 and day‐10 without reseeding; but reseeded to 1 × 10^5^ cells per mL on day‐7.

### 5.5 Flow cytometry

The cells were labeled with the following antibodies: anti‐human IgD, FITC (eBioscience™ 11986842; Invitrogen, USA); anti‐human IgG, PE (Thermo Scientific™ MA110377; Invitrogen, USA); anti‐human CD95, PE/Cyanine7 (305622; BioLegend, USA); anti‐human CD38, APC (eBioscience™ 17038942; Invitrogen, USA); anti‐human CD27, AlexaFluor™ 700 (eBioscience™ 56027942; Invitrogen, USA); and anti‐human CD40, PE (eBioscience™ 12040942; Invitrogen, USA). All centrifugation steps for washing were performed at 300 G for 10 min. The cells were analyzed via flow cytometer (Attune NxT Flow Cytometer, Thermo Fisher Scientific, USA), usually on days‐0, 4, 7, 10, and 13 of cultures. Flow cytometry data were analyzed using FlowJo (Version 10, BD, USA).

### 5.6 Image acquisition

All images were acquired using EVOS FL Auto imaging system (Thermo Fisher, USA) at 10×, 20×, and 63× magnification. Images were analyzed using ImageJ software (National Institute of Health, USA).

### 5.7 Functional assay on ASCs using ELISA

Naïve B‐cells were activated using indicated CD40L, BCR signaling factors, TLR9 agonist, BAFF, and ILs (IL‐2, ‐4, ‐10, ‐21) until day‐10. Cells were reseeded at 1 × 10^5^ cells per mL in 100 μL of the culture medium per well in 96‐well culture plates. Cells were incubated with media containing all factors except IL‐4 and BCR signaling factors for 2 days before the supernatants were harvested on day‐12 and frozen at −80°C. Immunoglobulin concentrations in the thawed supernatant were later determined using ELISA kits using p‐nitrophenyl phosphate as a substrate for alkaline phosphatase (Mabtech, Sweden). ELISA was performed in 96‐well High Binding Standard ELISA Microplates (Greiner, USA) using a standardized protocol. The media alone controls and serially diluted samples from each group were measured in triplicate. Plates were read using the Synergy HTX multi‐mode reader (BioTek, Agilent, USA) at 405 nm, after 20–60 min of incubation with the substrate.

### 5.8 Graphs and statistical analysis

All graphs were plotted and the statistical analysis was conducted using GraphPad Prism (Version 10, GraphPad, San Diego, USA). In general, one‐or two‐way ANOVA with Tukey's test was used to determine the significance of differences between the groups. For the comparison of two groups, the unpaired *t*‐test was used.

### 5.9 BCR repertoire sequencing

From the indicated culture conditions, >100,000 cells per sample were harvested on day‐13 of culture and stored in RLT buffer (RNeasy Kit, Qiagen). All sample preparation and sequencing steps were performed at iRepertoire, Inc. (Huntsville, AL). Briefly, the RNA was extracted from each sample and cDNA was prepared with an average input mass of 159 ng per reaction. Using a molecular identifier (MID) with dual indices, amplicon libraries of approximately 500 base pairs of human BCR Ig‐heavy chain genes were prepared. Upon washing and gel purification of amplicons, 527.5 ng of cDNA per sample was pooled into 1 run of 1 MID on the Illumina MiSeq using 500‐cycle kit and paired‐end read of 251 × 251 cycles. Each sample was run in duplicate.

### 5.10 BCR sequencing data analyses

All analyses on BCR sequencing data were performed using methods and algorithms developed by iRepertoire, Inc. The tree map was generated as an illustrative demonstration of the diversity of a BCR repertoire. In a tree map, the entire plot area is divided into sub‐areas according to V‐usage, which is then subdivided according to J‐usage, and then each unique CDR3 (uCDR3) within a given V‐J‐combination is subsequently represented by a rounded rectangle. The size of each rounded rectangle denotes the relative frequency of the particular V‐J‐uCDR3 sequence. The diversity of a BCR repertoire was also calculated by the DI, which is defined as 100 minus the area under the curve between the percentage of total reads and the percentage of unique CDR3s when unique CDR3s are sorted by frequency from largest to smallest. Assuming that $r_{1}\geq r_{2}\geq \cdots \geq r_{i}\geq r_{i+1}\geq \cdots \geq r_{n}$ where $r_{i}$ is the frequency of the *i*th CDR3 and *n* is the total number of unique CDRs, a plot of $x_{k}=\frac{k}{n}$ versus $y_{k}=\frac{\sum_{i=1}^{k}r_{i}}{\sum_{i=1}^{n}r_{i}}$ can be plotted. The DI can be calculated as 100 minus the area under the curve. The average frequency of SHM for each sample was calculated by counting all mismatches between a receptor sequence and its best IMGT V reference sequence, appearing from CDR1 to the end of V region, taking every unique sequence as an individual entry. The isotype UpSet plots were generated by showing all possible isotype combinations for each unique sequence as an individual entry.

## AUTHOR CONTRIBUTIONS

**Pearlson Prashanth Austin Suthanthiraraj:** Data curation; investigation; validation; formal analysis; visualization; writing – original draft. **Sydney Bone:** Data curation; investigation; validation; formal analysis; visualization. **Kyung‐Ho Roh:** Conceptualization; methodology; investigation; validation; formal analysis; supervision; funding acquisition; visualization; project administration; resources; writing – original draft; writing – review and editing; software.

## CONFLICT OF INTEREST STATEMENT

The authors have no conflicts of interest to declare.

## Supporting information


**Data S1:** Supporting Information

## Data Availability

The data that support the findings of this study are available from the corresponding author upon reasonable request.
