# Supplementary material for: Microbead‐based synthetic niches for in vitro expansion and differentiation of human naïve B‐cells
Source: Bioeng Transl Med. 2025 Jan 17;10(3):e10751. doi: 10.1002/btm2.10751 (PMC12079508; doi:10.1002/btm2.10751)
Supplement: Supplementary file 1 — Data S1: Supporting Information [file BTM2-10-e10751-s001.pdf]

Supplementary Information for

A microbead-based synthetic niche for in-vitro expansion and differentiation of human naïve B-cells

by

Pearlson Prashanth Austin Suthanthiraraj[1], Sydney Bone[1] and Kyung-Ho Roh[1,2,3,+]

[1]Department of Chemical and Materials Engineering, [2]Biotechnology Science and Engineering Program, [3]Materials Science Program, The University of Alabama in Huntsville, Huntsville, AL

[+]Corresponding Author

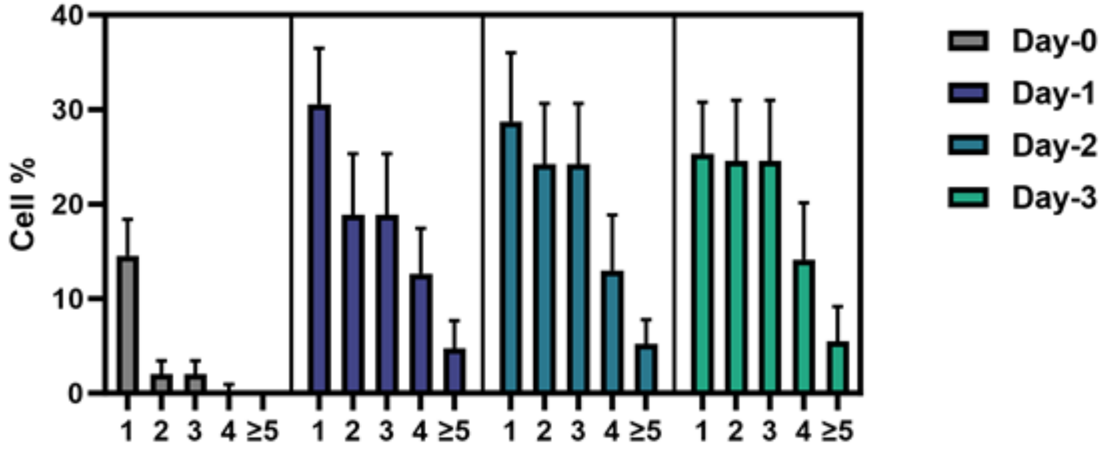

SI Figure 1. Beads-per-cell characterization for MB-CD40L

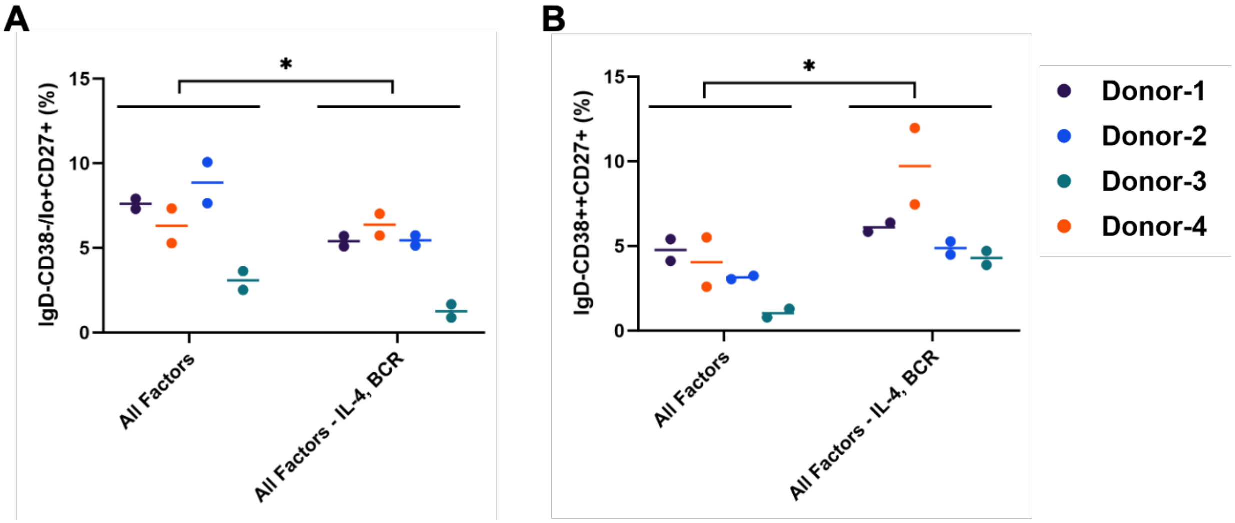

SI Figure 2. Effects of switching interleukins for the terminal differentiation of naïve B-cells isolated from four different donors. Scatter plots show the Day-13 yields (%) of memory B-cells (A) and ASCs (B) derived by indicated conditions. "All Factors" indicates a continued activation using MB-CD40L, anti-BCR/CD21 antibodies, TLR-agonist, and all IL-2, IL-4, IL-10, and IL-21 between Day-10 and Day-13. "All Factors – IL-4, BCR" indicates that the cells were cultured with all activating factors except IL-4 and anti-BCR/CD21 antibodies between Day-10 and Day-13. Statistical analysis was conducted using two-way ANOVA with Tukey's multiple comparisons (* $P<0.05$) to test the statistical significance of differences between the test groups.

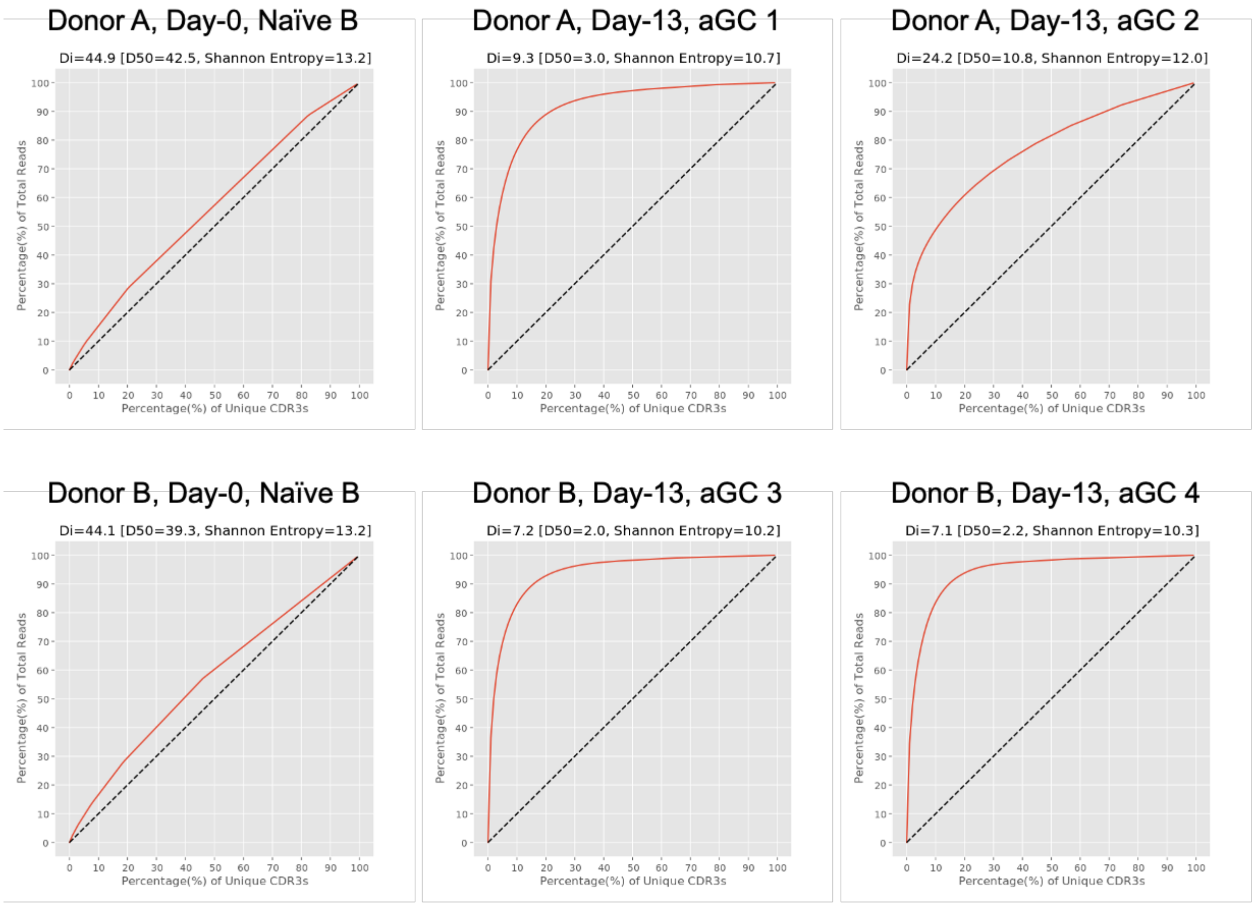

SI Figure 3. Diversity Index (DI) calculated for the naïve B-cells (Day 0) from two different donors and the artificial germinal center (aGC) B-cells (Day-13).

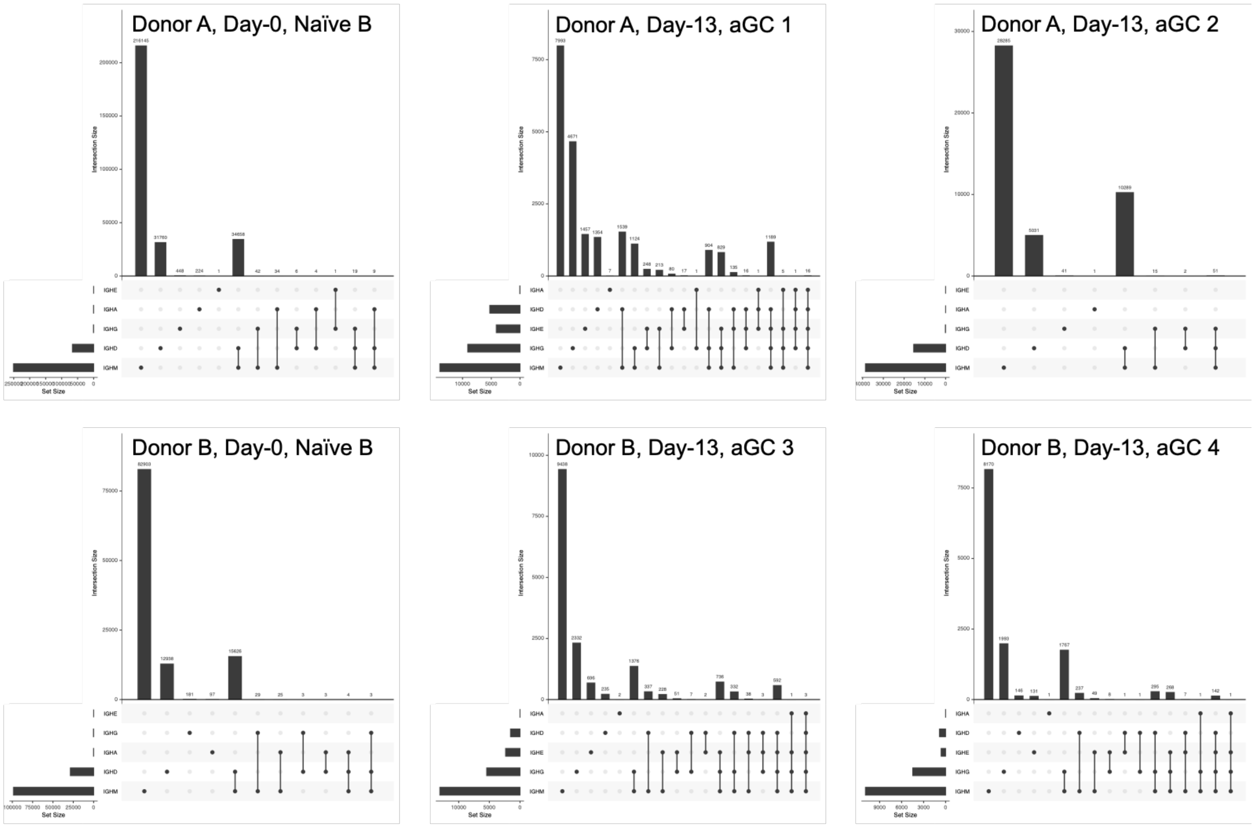

SI Figure 4. UpSet plots that show the frequencies of all detected isotypes and their combinations for unique IgV-gene sequences harvested from the naïve B-cells (Day 0) from two different donors and the artificial germinal center (aGC) B-cells (Day-13).
